# Assessment of compliance with hormonal therapy in early breast cancer patients with positive hormone receptor phenotype: A single institution study

**DOI:** 10.1016/j.breast.2022.01.017

**Published:** 2022-02-01

**Authors:** Shereef Ahmed Elsamany, Hossam Alghanmi, Abdelrahman Albaradei, Rasha Abdelhamid, Eman Madi, Amira Ramzan

**Affiliations:** a Oncology Center, King Abdullah Medical City, Makkah, Saudi Arabia; b Oncology Center, Mansoura University, Mansoura, Egypt; c King Abdullah Medical City, Makkah, Saudi Arabia; d Oncology Nurse, Oncology Center, King Abdullah Medical City, Makkah, Saudi Arabia

**Keywords:** Compliance, Adjuvant, Hormonal therapy, Breast cancer

## Abstract

**Background:**

Adherence to long-term adjuvant hormonal therapy in hormonal receptors (HR)-positive breast cancer is really challenging and can affect the survival outcome. The present study aims to assess rate of compliance with hormonal therapy and possible predictive factors in a single institute in Saudi Arabia.

**Patients &methods:**

We recruited patients with HR-positive breast cancer who presented to oncology outpatient clinics. Patients were assessed for compliance using a study questionnaire. Compliance was defined as taking ≥80% of prescribed doses of oral hormonal therapy. Different epidemiological, clinical, pathological and treatment data were checked in patients’ medical records and correlated with compliance/interruption of hormonal therapy.

**Results:**

Among the 203 recruited patients, 95.1% were compliant with hormonal therapy, while it was interrupted in 16.7% of patients, and 58.1% reported missing intake of hormonal pills. Age >50 years, having permanent job and higher education level were significantly associated with non-compliance in univariate analysis. On multivariate analysis, job status was the only independent predictor of non-compliance. The following parameters were significantly related to hormonal therapy interruption: marital status (single: 28.8% vs married patients: 12.6%, p = 0.01) and residence location (Makkah: 11.7% vs. outside Makkah: 25.3%, p = 0.019), lymphovascular invasion (LVI) (No: 20.9%, Yes: 7.8%, p = 0.025) and N0 tumours (compared to node-positive patients, p = 0.008). On multivariate analysis, marital status, residence location and N-stage, maintained significance relation with hormonal therapy interruption.

**Conclusion:**

Compliance with hormonal therapy was high in the study cohort. Marital status, residence location, job status and N-stage may be related to interruption/compliance with hormonal therapy.

## Highlights

• Compliance rate to adjuvant hormonal therapy was high in Saudi patients.
• Job status was significantly associated to hormonal therapy compliance.
• Marital status, residence location and N0-stage were linked with hormonal therapy interruption.
• Side effects were related to hormonal therapy interruption in a minority of patients.

## 1 Background

Breast cancer is the most common cancer among females worldwide and similarly, it ranks first among female cancers in Saudi Arabia [1,2]. Hormonal receptors (HR)-positive breast cancer constitutes about two thirds of breast cancer patients. Adjuvant hormonal therapy with/without chemotherapy is considered the standard of care in those patients [3,4]. Adjuvant hormonal therapy should continue for at least five years and even, several trials demonstrated improved survival outcome with extended adjuvant hormonal therapy beyond 5 years [[3], [4], [5], [6], [7], [8]]. In the ATLAS study, ten-years of tamoxifen decreased the risk of recurrence by 16% compared to 5 years [3]. Similar results were reported in the aTTom trial [4]. Furthermore, several trials demonstrated disease free survival benefit with extended use of aromatase inhibitors after 5 years of tamoxifen [5,6]. However, several adverse events have been reported with hormonal therapy and tolerance is highly variable among breast cancer patients [9]. Tamoxifen could produce hot flushes, vasomotor symptoms, vaginal dryness and impaired cognitive functions. Meanwhile, aromatase inhibitors are usually linked with musculoskeletal/joint pain, osteoporosis and elevated blood lipid levels [9,10].

Given the long-term duration over years, compliance to hormonal therapy is a critical issue and several studies had reported compromised survival outcome in patients with poor compliance less than 80% of the intended dose of hormonal therapy [10].

Noteworthy, several reports highlighted that only 50% of patients can successfully complete 5 years of hormonal therapy, however, the reported adherence rates are highly variable among different studies [10,11]. Several factors have been linked with poor compliance such as extremes of age, side effects, perception of low recurrence risk, poor patient-physician communication and lack of social support [10,11].

The present study aims to assess compliance to adjuvant hormonal therapy in HR-positive breast cancer patients treated in oncology centre, King Abdullah Medical City, Makkah, Saudi Arabia in addition to checking different factors associated with compliance/interruption of adjuvant hormonal therapy.

## 2 Method

### 2.1 Study population

We recruited patients with HR-positive breast cancer who presented to oncology outpatient clinics at King Abdullah Medical City, Makkah, Saudi Arabia within 6 months from time of study approval by the institutional review board. Recruited patients must have no evidence of metastasis at the time of diagnosis and underwent curative intent-breast surgery. They must have received adjuvant hormonal therapy for at least two years. Prior (neo)adjuvant chemotherapy, trastuzumab and radiotherapy were allowed as indicated by standard guidelines.

In this study, recruited patients were assessed for compliance with hormonal therapy through direct interview using a study questionnaire including potential epidemiological and personal/social factors correlated with the rate of compliance to hormonal therapy and hormonal therapy interruption. In addition, different clinical and pathological data were checked in patients’ medical records.

### 2.2 Definition of compliance

Patients were asked if they had interruption of hormonal therapy for successive days and if so, duration of interruption was checked and reported as follows: less than one week/month, one week to one month or more than one month. They were asked as well for incidental missing of pills on separate days and if happened, were asked for frequency of missing their pills to be reported as follows: rarely (≤once/month), infrequent (2–6 times/month), frequent (>6 times/month). Compliance to hormonal therapy is defined as taking ≥80% of prescribed doses of adjuvant hormonal therapy, which is assumed to be comparable to no interruption of hormonal therapy/no missing pills or interruption/missing pills less than one week/month in total.

### 2.3 Study methods

The study questionnaire included the following information: educational level, marital status, desire of pregnancy, job status, type of adjuvant hormonal therapy, any interruption of hormonal therapy for successive days, frequency of missing pills on separate days, receiving chemotherapy, if any, possible side effect from hormonal treatment.

In addition, the following data were retrieved from patients’ medical records: age at diagnosis, menopausal status, type and date of surgery, pathological T-stage, number of positive lymph nodes (LNs)/N-stage, tumour grade, lymphovascular invasion (LVI), ER status; percentage and staining intensity, PR status; percentage and staining intensity, HER2 status by immunehistochemistry (IHC) with FISH confirmation in cases with (++)by IHC. In addition, data of tumour relapse (if any) and dates of relapses were assessed as well.

### 2.4 Statistical analysis

We used SPSS version 17 statistical program. Categorical data were presented as percentages. Different epidemiological, treatment and clinicopathological factors were correlated with compliance and interruption of hormonal therapy using chi square test. Factors with significant correlation in univariate analysis were assessed using logistic regression to look for independent predictors for compliance or hormonal therapy interruption. Disease free survival (DFS) was assessed in patients with hormonal therapy interruption compared to those with no interruption using log-rank test. Hazard ratio was assessed using Cox regression method. As a general rule, an alpha value of 0.05 will be set a priori for all two tailed comparisons.

## 3 Results

We recruited 203 patients who were eligible to the study within the specified timelines. All patients were ER-positive except one (who was ER-negative/PR-positive), 74.9% were PR-positive and 24.6% were HER2-positive. Recruited patients received tamoxifen (34%), tamoxifen plus LHRH-analogue (5.9%), tamoxifen followed by an aromatase inhibitor (6.4%), aromatase inhibitor (41.9%) and aromatase inhibitor plus LHRH-analogue (11.8%). The recruited cohort included 2 males, perimenopausal (2%), premenopausal (47.8%) and postmenopausal females (49.2%). Regarding compliance with hormonal therapy, 95.1% were compliant with hormonal therapy (received ≥80% of the prescribed pills). Meanwhile, hormonal therapy was interrupted in 16.7% of patients. The duration of interruption was less than 1 week in 70.6%, one week to one month in 20.6% and more than one month in 8.8% of patients. Meanwhile, side effects from hormonal therapy were reported in 70.9% of patients. However, 59.1% of patients reported that it was well tolerated. Among those who reported hormonal therapy interruption, side effects related to hormonal therapy and logistic barriers were the cause of interruption in 11.8% of patients each. Meanwhile, low recurrence-risk perception was the reason of interruption in 5.9% of patients. Noteworthy, majority of patients reported other reasons of interruption (70.6%) such as forgetting the pills or travelling to a trip without taking the pills. Furthermore, 58.1% of patients reported missing intake of hormonal pills. Noteworthy, 95.8% of those patients reported that they rarely missed the pills (≤once/month) (Table 1).

**Table 1:** Interruption/missing of hormonal therapy.

Parameter		Frequency (n)	Percentage (%)
Interruption	Yes	34	16.7
	No	169	83.3
	Total	203	100.0
Duration of interruption	<1 week	24	70.6
	1 week-1 month	7	20.6
	>1 month	3	8.8
	Total	34	100.0
Cause of interruption	Side effects	4	11.8
	Logistics/availability	4	11.8
	Perception of no need	2	5.9
	Others	24	70.6
	Total	34	100.0
Missing pills	Yes	118	58.1
	No	85	41.9
	Total	203	100.0
Frequency of missing pills	Rarely (≤once/month)	114	96.6
	Infrequent (2–6 times/month)	3	2.5
	Frequent (>6 times/month)	1	0.9
	Total	118	100.0

### 3.1 Hormonal therapy interruption

Baseline clinicopathological characteristics were balanced in those who reported hormonal therapy interruption vs. no interruption except for LVI and N-stage where interruption was more common in those with no LVI (No: 20.9%, Yes: 7.8%, p = 0.025) and N0 (compared to N-positive patients, p = 0.008) (Table 2).

**Table 2:** Baseline tumour factors and relation to hormonal therapy interruption.

Parameters		Hormonal therapy interruption		Total	P-value
		Yes	No		
Pathological type	Invasive ductal	25	141	166	0.29
		15.1%	84.9%	100.0%	
	Invasive lobular	8	22	30	
		26.7%	73.3%	100.0%	
	Others	1	6	7	
		14.3%	85.7%	100.0%	
Site	Left	18	83	101	0.89
		17.8%	82.2%	100.0%	
	Right	15	82	97	
		15.5%	84.5%	100.0%	
	Bilateral	1	4	5	
		20.0%	80.0%	100.0%	
Pathological T-stage	Tx	1	4	5	0.96
		20.0%	80.0%	100.0%	
	T0	1	9	10	
		10.0%	90.0%	100.0%	
	T1	12	63	75	
		16.0%	84.0%	100.0%	
	T2	15	71	86	
		17.4%	82.6%	100.0%	
	T3	5	20	25	
		20.0%	80.0%	100.0%	
	T4	0	2	2	
		0.0%	100.0%	100.0%	
Multicentricity	Yes	3	24	27	0.58
		11.1%	88.9%	100.0%	
	No	31	145	176	
		17.6%	82.4%	100.0%	
Lymphovascular invasion	Yes	5	59	64	0.025
		7.8%	92.2%	100.0%	
	No	29	110	139	
		20.9%	79.1%	100.0%	
Grade	Grade 1	6	27	33	0.72
		18.2%	81.8%	100.0%	
	Grade II	22	100	122	
		18.0%	82.0%	100.0%	
	Grade III	5	28	33	
		15.2%	84.8%	100.0%	
	Unknown	1	14	15	
		6.7%	93.3%	100.0%	
N- stage	Nx	3	1	4	0.008
		75.0%	25.0%	100.0%	
	N0	20	70	90	
		22.2%	77.8%	100.0%	
	N1	5	59	64	
		7.8%	92.2%	100.0%	
	N2	4	30	34	
		11.8%	88.2%	100.0%	
	N3	2	9	11	
		18.2%	81.8%	100.0%	
PR status	Negative	6	45	51	0.39
		11.8%	88.2%	100.0%	
	Positive	28	124	152	
		18.4%	81.6%	100.0%	

Table 3 displays different parameters that may be related to interruption of hormonal therapy. The following parameters were significantly related to hormonal therapy interruption: marital status (single 28.8% vs. married patients 12.6%, p = 0.01) and residence location (Makkah 11.7% vs. outside Makkah 25.3%, p = 0.019). Meanwhile, no difference in incidence of hormonal therapy-interruption according to development of side effects (Yes:18.1% vs. No:13.6%, p = 0.54). On multivariate analysis including marital status, residence location, LVI and N-stage, all parameters maintained significance relation with hormonal therapy interruption except for LVI (Table 4).

**Table 3:** Relation of different epidemiological, tumour and treatment factors with hormonal therapy interruption.

		Hormonal interruption		Total (n = 203)	P-value
		Yes (n = 34)	No (n = 169)		
Marital status	Single	15	37	52	0.01
		28.8%	71.2%	100.0%	
	Married	19	132	151	
		12.6%	87.4%	100.0%	
Pregnancy desire	Yes	1	4	5	0.60
		20.0%	80.0%	100.0%	
	No	33	165	198	
		16.7%	83.3%	100.0%	
Education level	Not educated	12	51	63	0.56
		19.0%	81.0%	100.0%	
	Up to high school	15	92	107	
		14.0%	86.0%	100.0%	
	University level	5	22	27	
		18.5%	81.5%	100.0%	
	Postgraduate level	2	4	6	
		33.3%	66.7%	100.0%	
Work impact	No impact	29	144	173	1.0
		16.8%	83.2%	100.0%	
	Work impact	5	25	30	
		16.7%	83.3%	100.0%	
Side effects of hormonal therapy	Yes	26	118	144	0.54
		18.1%	81.9%	100.0%	
	No	8	51	59	
		13.6%	86.4%	100.0%	
Number of side effects	None	8	51	59	0.55
		13.6%	86.4%	100.0%	
	Single	13	69	82	
		15.9%	84.1%	100.0%	
	Multiple	13	49	62	
		21.0%	79.0%	100.0%	
Tolerance to hormonal therapy	Well tolerated	17	103	120	0.26
		14.2%	85.8%	100.0%	
	Moderately/poorly tolerated	17	66	83	
		20.5%	79.5%	100.0%	
Menopausal status	Premenopausal	15	82	97	0.84
		15.5%	84.5%	100.0%	
	Postmenopausal	18	82	100	
		18.0%	82.0%	100.0%	
	Perimenopausal	1	3	4	
		25.0%	75.0%	100.0%	
	Male	0	2	2	
		0.0%	100.0%	100.0%	
Chemotherapy	Yes	26	134	160	0.08
		16.3%	83.8%	100.0%	
	No	8	35	42	
		19.0%	81.0%	100.0%	
Hormonal therapy type	Tamoxifen	17	64	81	0.25
		21.0%	79.0%	100.0%	
	Aromatase inhibitor (AI)	17	105	122	
		13.9%	86.1%	100.0%	
HER2 status	Negative	24	129	153	0.52
		15.7%	84.3%	100.0%	
	Positive	10	40	50	
		20.0%	80.0%	100.0%	
Job status	No job	28	147	175	0.17
		16.0%	84.0%	100.0%	
	Temporary Job	0	7	7	
		0.0%	100.0%	100.0%	
	Permanent job	6	15	21	
		28.6%	71.4%	100.0%	
Residence	Makkah	15	113	128	0.019
		11.7%	88.3%	100.0%	
	Outside Makkah	19	56	75	
		25.3%	74.7%	100.0%	
Age at diagnosis	≤50 years	7	57	64	0.16
		10.9%	89.1%	100.0%	
	>50 years	27	112	139	
		19.4%	80.6%	100.0%

**Table 4:** Multivariate analysis of factors related to hormonal therapy interruption and compliance.

Interruption of hormonal therapy					
Parameters	Odd's Ratio	95% Confidence Interval			P-value
		Lower Bound		Upper Bound	
Marital status	3.24	1.40		7.48	0.006
Residence	0.42	0.19		0.91	0.029
Lymphovascular invasion	0.49	0.17		1.46	0.20
N-stage	2.93	1.23		6.98	0.015
Hormonal therapy compliance					
Parameters	Odd's Ratio	95% Confidence Interval			P-value
		Lower Bound	Upper Bound		
Job status	0.32	0.11	0.95		0.041
Age_	0.00	0.00	----.		0.99
Education level	0.92	0.32	2.64		0.87

### 3.2 Compliance with hormonal therapy

Regarding compliance with hormonal therapy, Age >50 years (p = 0.028), having permanent job (p = 0.006) and higher education level (p = 0.007) were significantly associated with non-compliance to adjuvant hormonal therapy in univariate analysis (Table 5). In addition, there was a trend towards increased non-compliance with hormonal therapy in single patients (p = 0.07) and those who reported multiple side effects (p = 0.068). On multivariate analysis using logistic regression including job status, age and educational level, job status was the only independent predictor of non-compliance (p = 0.011) (Table 4).

**Table 5:** Relation of epidemiological, tumour and treatment factors with compliance to hormonal therapy.

Parameters		Compliance		Total (n = 203)	P-value
		No (n = 10) 4.9%	Yes (n = 193) 95.1%		
Marital status	Single	5	47	52	0.07
		9.6%	90.4%	100.0%	
	Married	5	146	151	
		3.3%	96.7%	100.0%	
Pregnancy desire	Yes	0	5	5	0.61
		0.0%	100.0%	100.0%	
	No	10	188	198	
		5.1%	94.9%	100.0%	
Education level	Not educated	2	61	63	0.007
		3.2%	96.8%	100.0%	
	Up to high school	6	101	107	
		5.6%	94.4%	100.0%	
	University level	0	27	27	
		0.0%	100.0%	100.0%	
	Postgraduate level	2	4	6	
		33.3%	66.7%	100.0%	
Work impact	No impact	9	164	173	0.66
		5.2%	94.8%	100.0%	
	Work impact	1	29	30	
		3.3%	96.7%	100.0%	
Side effects of hormonal therapy	Yes	7	137	144	0.94
		4.9%	95.1%	100.0%	
	No	3	56	59	
		5.1%	94.9%	100.0%	
Number of side effects	No Side effects	3	55	59	0.068
		5.2%	94.8%	100.0%	
	Single	1	81	82	
		1.2%	98.8%	100.0%	
	Multiple	6	56	62	
		9.7%	90.3%	100.0%	
Tolerance to hormonal therapy	Well tolerated	6	114	120	0.95
		5.0%	95.0%	100.0%	
	Moderately/poorly tolerated	4	79	83	
		4.8%	95.2%	100.0%	
Menopausal status	Premenopausal	3	94	97	0.21
		3.1%	96.9%	100.0%	
	Postmenopausal	6	94	100	
		6.0%	94.0%	100.0%	
	Perimenopausal	1	3	4	
		25.0%	75.0%	100.0%	
	Male	0	2	2	
		0.0%	100.0%	100.0%	
Chemotherapy	Yes	8	152	160	0.97
		5.0%	95.0%	100.0%	
	No	2	41	43	
		4.8%	95.2%	100.0%	
		13.9%	86.1%	100.0%	
HER2 status	Negative	7	146	153	0.71
		4.6%	95.4%	100.0%	
	Positive	3	47	50	
		6.0%	94.0%	100.0%	
Job status	No job	6	169	175	0.006
		3.4%	96.6%	100.0%	
	Temporary Job	0	7	7	
		0.0%	100.0%	100.0%	
	Permanent job	4	17	21	
		19.0%	81.0%	100.0%	
Residence	Makkah	4	124	128	0.12
		3.1%	96.9%	100.0%	
	Outside Makkah	6	69	75	
		8.0%	92.0%	100.0%	
Age	≤50	0	64	64	0.028
		0.0%	100.0%	100.0%	
	>50	10	129	139	
		7.2%	92.8%	100.0%	
Pathological T-stage	Tx	0	5	5	0.48
		0.0%	100.0%	100.0%	
	T0	0	10	10	
		0.0%	100.0%	100.0%	
	T1	2	73	75	
		2.7%	97.3%	100.0%	
	T2	5	81	86	
		5.8%	94.2%	100.0%	
	T3	3	22	25	
		12.0%	88.0%	100.0%	
	T4	0	2	2	
		0.0%	100.0%	100.0%	
Grade	Grade 1	1	32	33	0.58
		3.0%	97.0%	100.0%	
	Grade II	8	114	122	
		6.6%	93.4%	100.0%	
	Grade III	1	32	33	
		3.0%	97.0%	100.0%	
	Unknown	0	15	15	
		0.0%	100.0%	100.0%	
Lymphovascular invasion (LVI)	Yes	1	63	64	0.13
		1.6%	98.4%	100.0%	
	No	9	130	139	
		6.5%	93.5%	100.0%	
Multicentricity	Yes	1	26	27	0.75
		3.7%	96.3%	100.0%	
	No	9	167	176	
		5.1%	94.9%	100.0%	
N-stage	N0	6	88	94	0.37
		6.4%	93.6%	100.0%	
	N-positive	4	105	109	
		3.7%	96.3%	100.0%	
Hormonal therapy type	Tamoxifen	5	64	69	0.55
		7.2%	92.8%	100.0%	
	Aromatase inhibitor (AI)	4	81	85	
		4.7%	95.3%	100.0%	
	Tamoxifen + AI	0	13	13	
		0.0%	100.0%	100.0%	
	LHRH + Tamoxifen	1	11	12	
		8.3%	91.7%	100.0%	
	LHRH + AI	0	24	24	
		0.0%	100.0%	100.0%	

### 3.3 Survival outcome

**Fig. 1:**
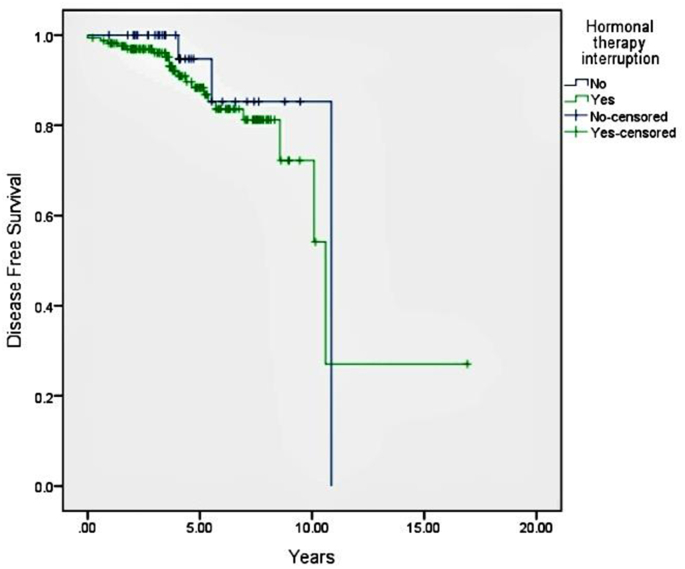
Disease free survival according to hormonal therapy interruption.

After a median follow up of 49 months, there was no statistically significant difference in DFS between patients who reported no hormonal therapy interruption compared to those with interruption (5-year DFS 94.7% vs. 88.4%, respectively, HR 0.62, 95% confidence interval 0.47–5.58, p = 0.44) (Fig. 1).

## 4 Discussion

Compliance with long-term adjuvant hormonal therapy is increasingly a major concern in patients with HR-positive breast cancer. In a meta-analysis of adjuvant hormonal studies with tamoxifen or aromatase inhibitor, 23%–28% of patients stopped hormonal therapy before completing the intended duration. However, higher rate of treatment discontinuation was reported in patients outside clinical trials (30%–50%) [13].

Several reports displayed that non-compliance with hormonal therapy can increase the risk of relapse and compromise the survival outcome of those patients. For example, in a study including 1633 patients treated with adjuvant tamoxifen, better treatment compliance was associated with improved survival outcome compared to patients with adherence <80% [14]. Lack of significant DFS difference according to hormonal therapy interruption in our study may be related to the small sample size of the study group.

Noteworthy, several methods of assessment of patient adherence have been used in different studies; however, these strategies have several shortcomings, which make assessment of adherence challenging. Furthermore, this may complicate comparison of results of different studies. Most of the studies used patient's self-reported questionnaire, which is limited by inaccurate reporting and tendency of patients to overestimate their compliance with hormonal therapy [15,16]. Other studies checked pharmacy/prescription records, which usually reveal lower compliance rate compared to that of self-reported questionnaires [17].

In the present study, compliance rate was high among recruited patients. The majority of patients reported interruption for less than one week and it was mostly related to forgetting the pills. Almost two thirds of patients reported that hormonal therapy was well tolerated; that is why side effects was not a major cause of hormonal therapy interruption in the study group despite being reported by the majority of patients. We explored different social, epidemiological and clinicopathological factors that may affect compliance with adjuvant hormonal therapy. Hormonal therapy interruption was less likely in married patients, those who live in the same location of the treating hospital and those with node-positive disease, while job status was the only independent predictor of non-compliance to hormonal therapy.

Increased probability of non-compliance of node-negative patients may reflect the perception of low risk of relapse and lack of necessity of adjuvant hormonal therapy. In contrast, in a study involving 161 patients treated with tamoxifen or aromatase inhibitors, patients with N2 disease and HER2 positivity were linked with non-adherence to hormonal therapy [18]. Living in remote locations may decrease the access to medical care and compromise re-dispensing of hormonal therapy. This was especially relevant in the era of COVID-19 pandemic with repeated lock-down and travel restrictions.

In our study, no difference in the compliance/interruption of hormonal therapy rate according to the type of hormonal therapy. Meanwhile, in a small study that included 89 patients (50 on tamoxifen and 39 on anastrozole), Ziller et al. reported that 80% of patients on tamoxifen and 69% on anastrozole have received the intended duration of adjuvant hormonal therapy [17]. Similar to our study, Ziller et al. used self-reported questionnaire; however, they had a medical record review as well as recall of prescription details by physicians. However, that study is limited by the small sample size and potential inaccuracy of assessment methods including physicians’ recall [17]. Moreover, prescription of hormonal therapy is not necessarily linked with pills intake by the patients. Differences in sample size, study design and methods of adherence assessment may account for discrepancy of the results compared to our study.

We found no difference in compliance rate according to age, menopausal status or chemotherapy. However, in a study including 2378 early stage breast cancer patients, adherence rates were lower in young (<45 years) and very old (≥85 years) patients [19]. Similar finding was detected in several other studies as well [[19], [20], [21]]. Meanwhile, hormonal therapy compliance was assessed in a Swedish study including patients with HR-positive breast cancer patients registered in the Swedish breast cancer registry, between 2009 and 2012. Compliance was evaluated using medication possession ratio (MPR), defined as the days’ supply of medication during the period from the first dispensing till the last dispensing in the time period (3 and 5 years), divided by number of days. Adherence rate was 91.2% and 91.5% after 3 and 5 years, respectively [20]. Noteworthy, studies based on registries/databases which have even similar designs, report a wide range of adherence ranging from 60 to 82% and 46–73% after 3 and 5 years, respectively [[22], [23], [24]]. No difference in compliance according to age and other parameters such as type of hormonal therapy and chemotherapy [20].

Similarly, previous studies reported lower adherence rate in those who developed side effects from hormonal therapy [18]. Noteworthy, there was a trend towards higher rate of non-compliance in patients with multiple side effects in our study. Variation in study designs, methods of assessment, sample size and limitations of the assessment methods may account for variation of these results.

Noteworthy, our study has some strengths such as assessment of the relation of several parameters that were not assessed in previous studies such as desire of pregnancy, educational level, job status, impact of hormonal therapy on work and tolerance of hormonal therapy. In addition, several tumour and treatment related factors were assessed as well. Our study is limited with the relatively small sample size, variation of the duration of adjuvant hormonal therapy among study participants and the limitations of assessment of compliance with self-reported questionnaire.

In conclusion, compliance with hormonal therapy was high among the study participants which may reflect over-estimation of compliance by patients’ self-reported questionnaire. Some social/epidemiological parameters such as marital status, job status and location of residence may affect compliance/interruption of adjuvant hormonal therapy.

## Ethical statement

-All procedures performed in studies involving human participants were in accordance with the ethical standards of the institutional and/or national research committee and with the 1964 Helsinki declaration and its later amendments or comparable ethical standards.

-For this study formal consent was not required.

## Declaration of competing interest

-Shereef Elsamany:

Roche: honoria of lectures, advisory boards.

Novartis: honoria of lectures, advisory boards.

Pfizer: honoria of lectures, advisory boards.

Amgen: research grant, honoria of lectures, advisory boards.

MSD: honoria of lectures, advisory boards.

-Other co-authors: no conflict of interest.
